# Time of flight dual photon emission computed tomography

**DOI:** 10.1038/s41598-020-76526-z

**Published:** 2020-11-11

**Authors:** Chih-Chieh Chiang, Chun-Chao Chuang, Yu-Ching Ni, Meei-Ling Jan, Keh-Shih Chuang, Hsin-Hon Lin

**Affiliations:** 1 Medical Physics Research Center, Institute for Radiological Research, Chang Gung University/Chang Gung Memorial Hospital, Taoyuan, Taiwan; 2 Department of Biomedical Engineering and Environmental Sciences, National Tsing-Hua University, Hsinchu, Taiwan; 3 Department of Medical Imaging and Radiological Sciences, Chung Shan Medical University, Taichung, Taiwan; 4 Department of Radiation Oncology, Chang Gung Memorial Hospital, Taoyuan, Taiwan; 5 Department of Nuclear Medicine, Keelung Chang Gung Memorial Hospital, Keelung, Taiwan; 6 Health Physics Division, Institute of Nuclear Energy Research, Atomic Energy Council, Taoyuan, Taiwan

**Keywords:** Medical imaging, Radionuclide imaging, Tomography

## Abstract

Time-of-flight dual photon emission computed tomography (TOF-DuPECT) is an imaging system that can obtain radionuclide distributions using time information recorded from two cascade-decay photons. The potential decay locations in the image space, a hyperbolic response curve, can be determined via time-difference-of-arrival (TDOA) estimations from two instantaneous coincidence photons. In this feasibility study, Monte Carlo simulations were performed to generate list-mode coincidence data. A full-ring positron emission tomography-like detection system geometry was built in the simulation environment. A contrast phantom and a Jaszczak-like phantom filled with Selenium-75 (Se-75) were used to evaluate the image quality. A TOF-DuPECT system with varying coincidence time resolution (CTR) was then evaluated. We used the stochastic origin ensemble (SOE) algorithm to reconstruct images from the recorded list-mode data. The results indicate that the SOE method can be successfully employed for the TOF-DuPECT system and can achieve acceptable image quality when the CTR is less than 100 ps. Therefore, the TOF-DuPECT imaging system is feasible. With the improvement of the detector with time, future implementations and applications of TOF-DuPECT are promising. Further quantitative imaging techniques such as attenuation and scatter corrections for the TOF-DuPECT system will be developed in future.

## Introduction

The time-of-flight (TOF) technique is commonly used with emission tomography, especially in positron emission tomography (PET). PET measures the two annihilation photons that are produced back-to-back after positron emission from a radionuclide. Traditional PET coincidence is used to determine along which line of response (LOR) an annihilation has occurred. TOF information can further confine approximately the position of annihilation along the line of annihilation based on the measured difference in arrival times. For the last several decades, extensive research has been performed on time-of-flight positron emission tomography (TOF-PET)^1,2^. It is well known that PET imaging incorporating TOF information can improve the signal-to-noise ratio (SNR) and achieve higher image quality^3^. The effect of TOF gain on the SNR improvement is proportional to the square root of the object size divided by the coincidence time resolution (CTR)^3^. The accuracy of the localization along the line of response can also be improved as the CTR decreases.

Other than dual photons caused by the pure electron–positron annihilation from positron emitters, many cascaded isotopes that emit a minimum of two photons per disintegration can simultaneously form one or more than two-photon detection in pairs^4,5^. The direction of successive γ-rays in a cascade has a small angular correlation, and it cannot explicitly provide the information of isotope position like the annihilation gammas. Traditionally, the emitted multiple photons for such isotopes are imaged using single-photon emission tomography (SPECT). To effectively exploit the timing characteristics of these isotopes, some studies have developed detection systems to reconstruct images using gamma–gamma coincidence data^6–9^. The coincidence imaging technique has also been implemented as an intraoperative probe to detect suspected tumor sites and sentinel lymph nodes^10,11^. Powell introduced a method that applies TOF information to localize the distribution of multiphoton emitters through collimators^12^. When photons in coincidence are detected, the source location can be determined as the intersection of line of response derived from collimated detector and hyperbola defined by the TOF information. However, the sensitivity of Powell’s system was too low due to the necessity of absorptive collimation. In addition, no imaging result was presented in his paper to demonstrate the viability of this concept.

As collimators have the most important restrictions in sensitivity for SPECT, a great step forward will be taken if a radiopharmaceutical spatial distribution in the body is obtained using a gamma camera without a collimator. In this study, we propose a time-of-flight dual-photon emission computed tomography (TOF-DuPECT) system to detect cascade photons without collimators and to reconstruct the distribution of radionuclides incorporating the time-difference-of-arrival (TDOA) technique^13^. The TDOA technique is a widely used passive source location technique for wireless communication and navigation systems^14,15^. For each measured TDOA, a hyperbola can be defined on which the emitter must lie. This concept is similar to the study proposed by Powell^12^. Here, we extend the concept to image reconstruction for the proposed TOF-DuPECT system. The knowledge of TOF difference provides independent spatial information on the source position and can be used for direct reconstruction of the dual photons using a hyperbola-of-response projector.

For the TOF-DuPECT system, a suitable image reconstruction algorithm is required to effectively obtain the radionuclide distribution. Commonly used iterative image reconstruction algorithms, such as maximum likelihood expectation maximization (MLEM) or ordered subset expectation maximization^16,17^, are difficult to apply to the TOF-DuPECT system owing to the geometrical complexity of the hyperbolic response curve and the heavy computational requirements. In recent years, the stochastic origin ensemble (SOE) algorithm has been used on nuclear medicine image reconstruction, especially for Compton cameras^18,19^ and PET imaging^20–22^. The SOE approach is based on the Monte Carlo Markov chain method incorporating the Metropolis–Hastings algorithm^23^. In general, the SOE algorithm is capable of obtaining similar results to the MLEM algorithm^18^ and its statistical derivation has been published^21^. When the reconstruction is based on list-mode (LM) data, the SOE algorithm can achieve higher efficacy than LM-MLEM because the forward- and back-projection operators are not computationally expensive during the iterative process^18^. However, note that the computation time is proportional to the size of the dataset. Overall, the SOE approach is an easy-to-use and efficient method with a low computational cost. Therefore, the SOE approach is well suited to our preliminary feasibility study of TOF-DuPECT.

In the TOF-DuPECT system, CTR dominates the localization accuracy and the reconstructed spatial resolution. Consequently, the system performance at various detector CTRs are considered and evaluated. For the performance evaluation, several image quality metrics are used, including the coefficient of variation (CV), contrast recovery coefficient (CRC), contrast-to-noise ratio (CNR), and spillover ratio (SOR).

## Methods

### TDOA Technique for TOF-DuPECT imaging

The imaging system for TOF-DuPECT is based on the concept of the TDOA location estimation technique (sometimes called the TOF technique). The TDOA technique is used to measure the relative difference between signal arrival times and to determine the location of an emitter in space^24^. Based on the TDOA technique, the TDOA of two detectors is given by

1 $$\mathrm{TDOA}=\frac{1}{v}\left({d}_{1}-{d}_{2}\right),$$

where *v* is the speed of light and *d*_1_ and *d*_2_ are the distances between the source and the two detectors. Equation (1) can be rewritten in the following form:

2 $$\left|{d}_{1}-{d}_{2}\right|=\Delta d=v\times \left|\mathrm{TDOA}\right|=\mathrm{constant},$$

which fulfills the requirement of a hyperbola. A hyperbola is defined as the locus of points where the absolute value of the difference of the distances to the two foci is constant. Specifically, a canonical form of a horizontally aligned hyperbola is given by

3 $$\frac{{(x-{x}_{0})}^{2}}{{a}^{2}}-\frac{{\left(y-{y}_{0}\right)}^{2}}{{b}^{2}}=1,$$

where (*x*_0_, *y*_0_) is the center of the hyperbola, *a* is the length of the semi-major axis, and *b* is the length of semi-minor axis. The parameter *b* is related to the distance between the foci, Δ*ℓ*, such that.

4 $$\Delta \ell=2\sqrt{{a}^{2}+{b}^{2}}.$$

An important property of a hyperbola is that the absolute value of the distance difference Δ*d* from each of the foci to a point on the hyperbola is constant, i.e.,

5 $$\Delta d=2a=\mathrm{constant}.$$

Therefore, introducing the Eq. (5) into Eq. (2), the parameter *a* then can be rewritten as Eq.

6 $$a=\frac{1}{2}v\left|\mathrm{TDOA}\right|,$$

The parameters *b*, and Δ*ℓ* can be also rewritten as

7 $$b=\sqrt{\frac{1}{4}{\Delta \ell}^{2}-{a}^{2}}=\frac{1}{2}\sqrt{({{x}_{2}-{x}_{1})}^{2}+{({y}_{2}-{y}_{1})}^{2}-{(v\times \mathrm{TDOA})}^{2}},$$

8 $$\Delta \ell=\sqrt{({{x}_{2}-{x}_{1})}^{2}+{\left({y}_{2}-{y}_{1}\right)}^{2}}.$$

Here, (*x*_1_, *y*_1_) and *(x*_2_, *y*_2_) are the coordinates of the two foci (the detectors).

**Figure 1:**
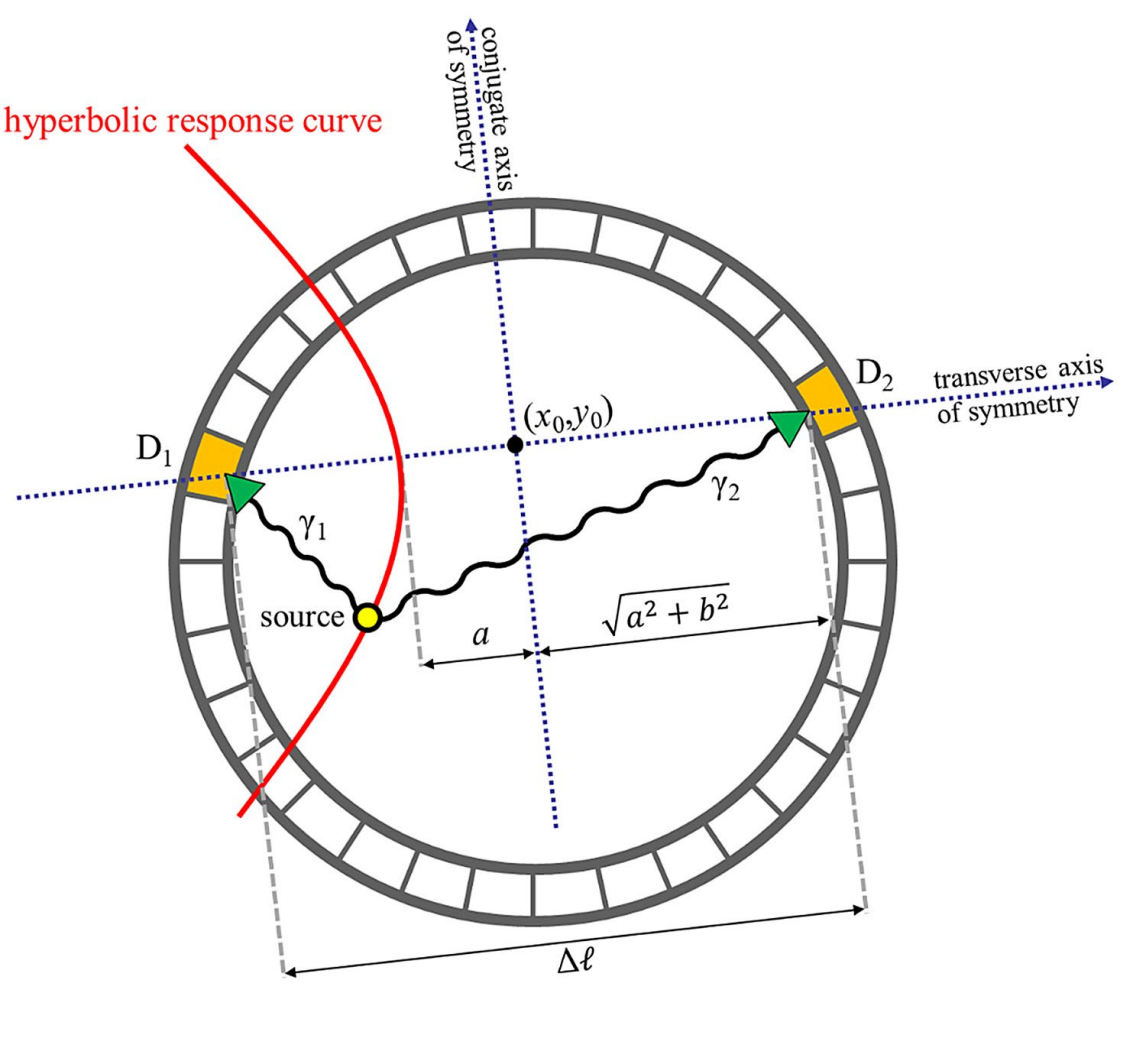
Illustration of the time-difference-of-arrival (TDOA) technique for TOF-DuPECT. The likelihood emitter position in space (locus of the hyperbola, red line) can be determined when the TDOA and the coordinates of the detectors (D_1_ and D_2_) are known.

Based on the above equations, the likelihood emitter positions can be determined when the TDOA information and the coordinates of the detectors are known. The hyperbolic response curve can then be directly determined in space, as illustrated in Fig. 1. Note that there is only one branch of the hyperbola that corresponds to the actual possible emitter position, depending on the sequential order of the arrival times of the two coincidence photons. The hyperbolic locus will be closer to the detector that receives the first arrival photon from the same decay cascade.

**Figure 2:**
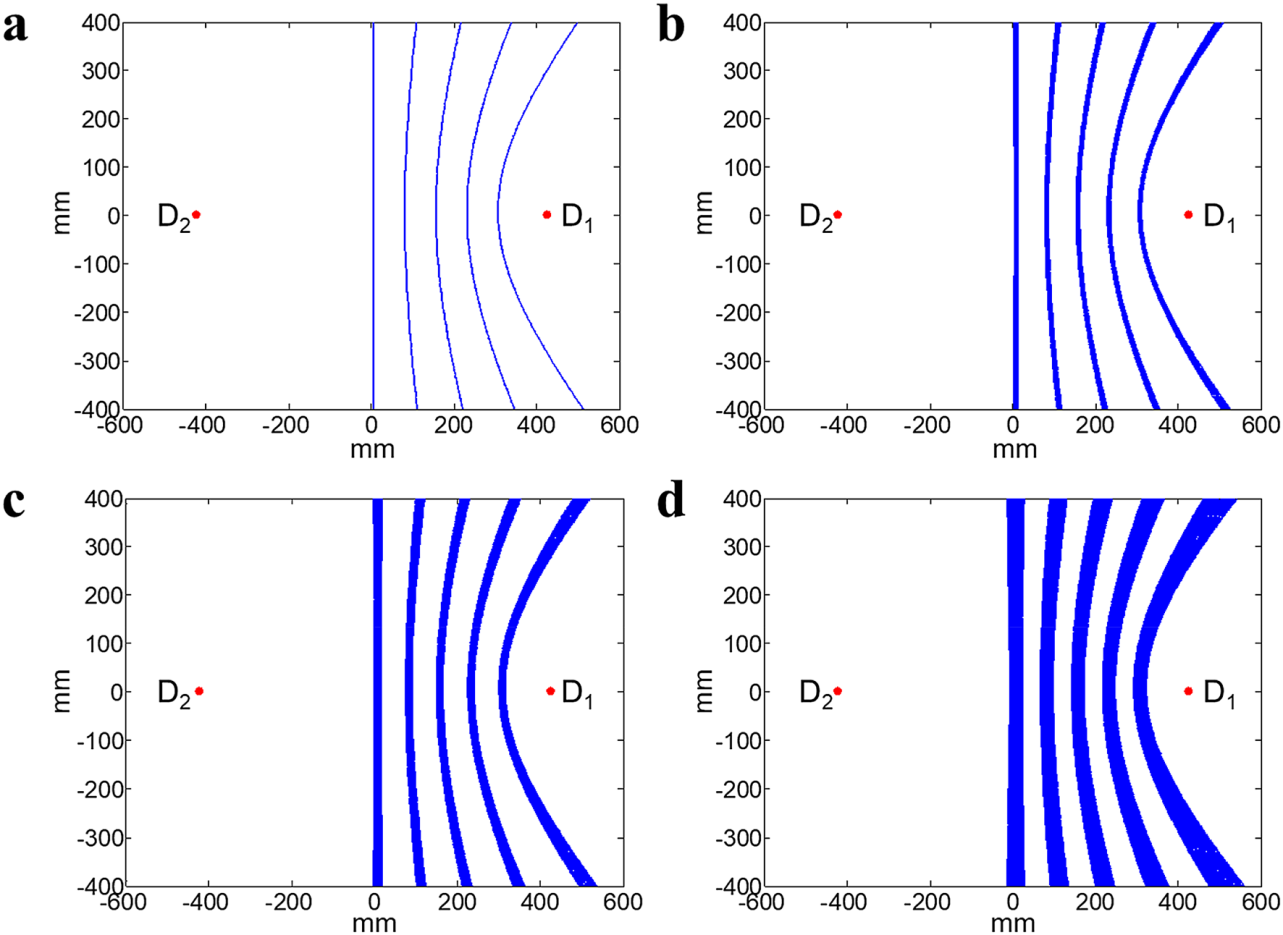
Hyperbolic response curves for CTR values of (**a**) 0 ps, (**b**) 50 ps, (**c**) 100 ps, and (**d**) 200 ps. From left to right in each panel, the hyperbolic response curves correspond to TDOAs of 0 ns, 0.5 ns, 1 ns, 1.5 ns, and 2 ns. D_1_ and D_2_ denote the two detectors that received dual photons, located at (424.5 mm, 0 mm) and (− 424.5 mm, 0 mm), respectively. Here, D_1_ is assumed to receive the first arrival photon from the same decay cascade.

Δ*d* corresponds to the difference between distances from the likelihood emitter positions along the hyperbola to the two detectors and is derived from TDOA. The timing uncertainty of a coincidence event along the hyperbolic response curve dominates the localization accuracy and is directly affected by the CTR of the detector. Figure 2 shows the hyperbolic response curves corresponding to TDOAs of 0 ns, 0.5 ns, 1 ns, 1.5 ns, and 2 ns for different CTRs. As can be seen, the localization uncertainty increases as the CTR increases. To investigate the imaging performance of the proposed system, CTRs of 0 ps, 50 ps, 100 ps, 150 ps, and 200 ps full width at half maximum (FWHM) were considered in this study.

### SOE reconstruction for the TOF-DuPECT system

As in a typical SOE algorithm^18,20^, the initial origin ensemble at the starting state *s*_0_ is generated by randomly selecting possible origins on the corresponding hyperbolic response curve inside the field of view (FOV) of the reconstructed domain for each recorded coincidence event *k*. The number of origins in a voxel *i* for a state *s* is denoted as the event density *c*_*i,s*_. The reconstruction procedure can be briefly described via the following steps:

(i) At the current state *s*, for the selected candidate event *k*, calculate the event density *c*_*i,s*_.
(ii) For the new state *s',* randomly select a new possible location corresponding to a new voxel *i'* for the candidate event *k* on its own corresponding hyperbolic response curve. The event density *c*_*i',s'*_ is then calculated.
(iii) Move the origin of the event *k* to the new candidate voxel *i'* with the transition acceptance probability *A* as follows:
  9 $${A}_{k(i\to {i}^{\mathrm{^{\prime}}})}\left(s\to {s}^{\mathrm{^{\prime}}}\right)=\mathrm{min}\left(1,\frac{\prod {s}^{\mathrm{^{\prime}}}}{\prod s}\right)=\mathrm{min}\left(1,\frac{{\alpha }_{k{i}^{\mathrm{^{\prime}}}}{\left({c}_{i,s}\right)}^{{c}_{i,s}}{\left({c}_{{i}^{\mathrm{^{\prime}}},{s}^{\mathrm{^{\prime}}}}+1\right)}^{{c}_{{i}^{\mathrm{^{\prime}}},{s}^{\mathrm{^{\prime}}}}+1}/{\varepsilon }_{{i}^{\mathrm{^{\prime}}}}}{{\alpha }_{ki}{\left({c}_{i,s}+1\right)}^{{c}_{i,s}+1}{\left({c}_{{i}^{\mathrm{^{\prime}}},{s}^{\mathrm{^{\prime}}}}\right)}^{{c}_{{i}^{\mathrm{^{\prime}}},{s}^{\mathrm{^{\prime}}}}}/{\varepsilon }_{i}}\right),$$
  where *α* denotes the probability that the event originates at the location for each event *k* and *ε* denotes the detection sensitivity at the voxel *i*. For simplicity, *α* and *ε* are set to 1.
(iv) Return to step (i) and repeat all the steps *N* times, where *N* is defined as the total number of detected coincidence events.

**Figure 3:**
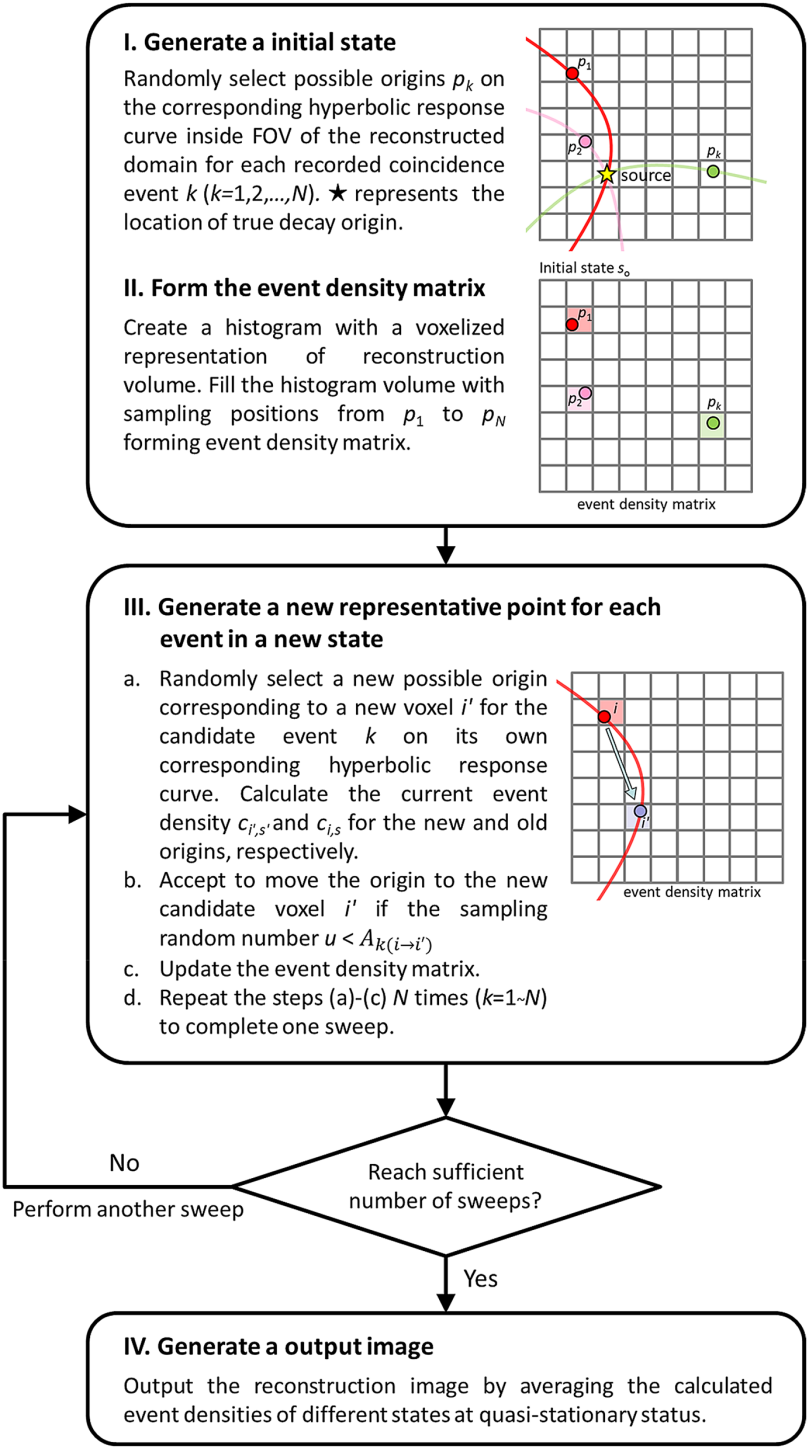
Flow chart of the SOE reconstruction algorithm for TOF dual photon imaging.

We define one sweep as *N* repetitions of the above steps. After a sufficient number of sweeps, the possible origins should converge to the actual position of the emitters and the equilibrium state should be reached. The OE (origin ensemble) average corresponding to the ensemble expectation is then obtained by averaging the measured event densities (*c*_*i,s*_) of different states at quasi-stationary status. The overall algorithm is illustrated in Fig. 3. In this study, there were 1500 sweeps for all of the experiments. The OE average constructed by averaging the last 300 sweeps were produced as the final output for the phantom studies. Note that the proposed algorithm is similar to a typical SOE algorithm for PET or a Compton camera. The major difference is that the possible location of the origin is always moving on the hyperbolic response curve corresponding the TDOA information during the iterative process.

### Dual-photon emitter

The dual-photon emitters are the radionuclides, which emit at least two photons in a cascade. Some researchers have indicated that there are radionuclides that can be used in gamma–gamma coincidence imaging^4,8^. However, while many radionuclides have this characteristic, not all are suitable for the proposed system. For TOF-DuPECT, the half-lives of the intermediate states of decay increase the uncertainty in the estimation of the possible location of the source; this is an important restriction on the usage of various radionuclides. In general, nuclides with intermediate states having half-lives on the order of a picosecond or less are required for our proposed system. While there is a restriction due to the decay characteristic, some radionuclides of interest having relatively short half-lives and suitable energies could be used, such as Se-75 (11.2 ps), Cr-48 (1 ns), K-43 (48 ps), and Co-60 (0.9 ps), where the values in parentheses indicate the half-lives of the intermediate states. In addition, a short half-life enables us to reduce the coincidence time window and suppress the effect of random coincidence.

**Figure 4:**
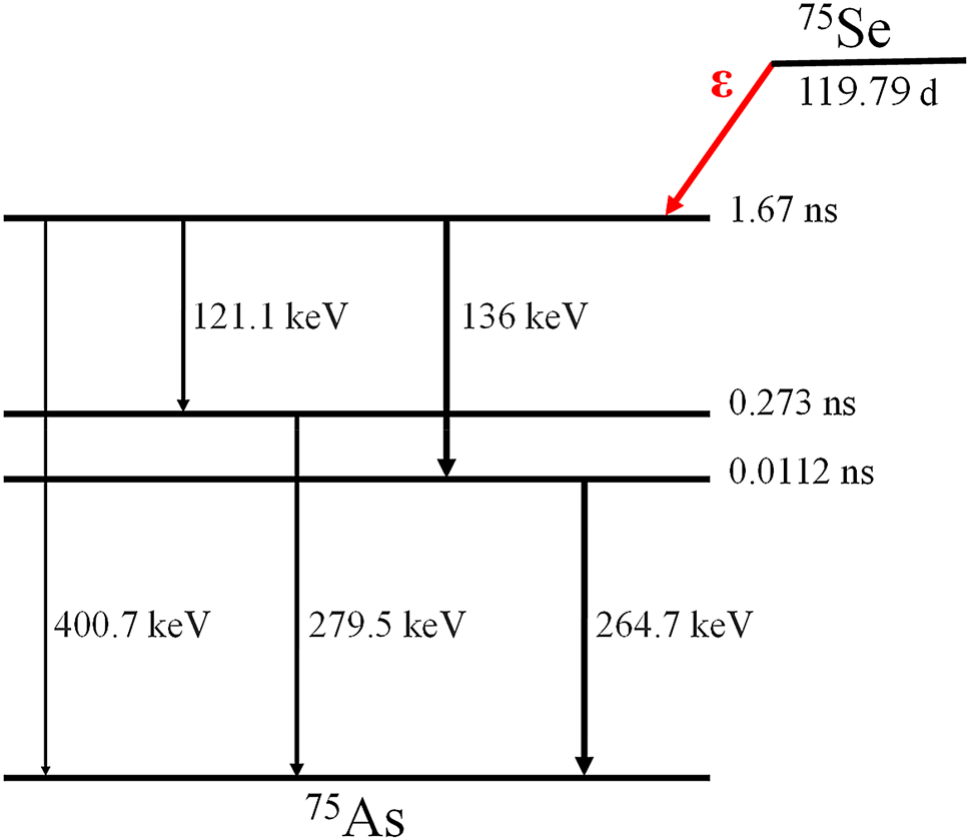
Simplified decay scheme for Se-75.

In this study, the dual-photon emitter used is Se-75, which has a half-life of 120 days and disintegrates 100% via electron capture to excited levels and to the ground state of As-75. Se-75 emits at four major emission energies of 121.1 keV, 136 keV, 264.7 keV, and 279.5 keV with half-lives of 273 ps and 11.2 ps at the 279.5-keV and 264.7-keV levels, respectively^25^. Figure 4 shows a summarized decay scheme for Se-75. In clinical applications, Se-75 selenomethionine, with sufficient pancreas specificity, is useful for pancreas scanning^26^. Se-75 homocholic acid taurine can be applied in gamma camera imaging to investigate bile acid malabsorption and to measure bile acid pool loss^27,28^.

### Monte Carlo simulations and data generation

To validate and evaluate the proposed TOF-DuPECT system, the Geant4 Application for Tomographic Emission (GATE) Monte Carlo package^29^ combined with a Simulation System for Emission Tomography (SimSET) multiple photon history generator^30^ was used to generate the LM coincidence data. To simulate a realistic nuclear decay process, the intermediate state half-life was considered in the simulation. The half-lives of each intermediate state of decay were modeled in the SimSET multiple photon history generator as in our previous study^31^. This hybrid Monte Carlo simulation code has also been used in a previous study of the DuPECT system^10^. Note that the cascade photon directions are assumed to be non-correlated in the simulation and that the photons emit isotropically from the radionuclide. The scatter and attenuation effects were not modeled in the simulation.

In the simulation, the TOF-DuPECT system was built in reference to the geometry of the Siemens Biograph 6 PET scanner^32^, which is a typical cylindrical PET system. The scanner has 48 detector modules arranged in three block rings. Each module consists of 13 × 13 lutetium oxyorthosilicate (LSO) crystals, and each crystal is 4 cm × 4 cm × 20 cm. In addition, the scanner has a transverse FOV of 58.5 cm and an axial FOV of 16.2 cm. Different energy resolutions were set between the minimum (12%) and maximum (18%) values referenced at 511 keV for each crystal in the detector module, and the detection efficiency factor was set to 0.9. To estimate the performance of the TOF-DuPECT system under various CTR conditions, five different detector CTRs from 0 to 200 ps in steps of 50 ps were considered for the proposed system. The output singles from the Monte Carlo simulation code were acquired in LM format. The pairs of coincident singles were registered within a time window of 2 ns and then sorted into a 2D dataset via single-slice rebinning with a ring maximum difference of ± 2, which assumes that oblique data can be assigned to non-oblique 2D transverse slices. A dual-energy window was used to discriminate coincidence events during the data acquisition process. The lower and higher energy windows were set to 108–164 keV and 211–318 keV, respectively. The sequential order of the arrival times of the two photons was used to reduce the random fraction.

### Point source measurement

A Se-75 point source with a total activity of 2 mCi was used in the experiment with an acquisition time of 10 s. The point source was placed at the center of the FOV to measure the system sensitivity and resolution under different CTR conditions. The system sensitivity is defined as the total counts of measured coincidence events per unit time of the source activity. In the measurement of spatial resolution under different CTRs, the widths of the point spread function were defined by its FWHM. All the images were reconstructed into a 64 × 64 matrix with a 4.5-mm pixel size.

### Phantom study

**Figure 5:**
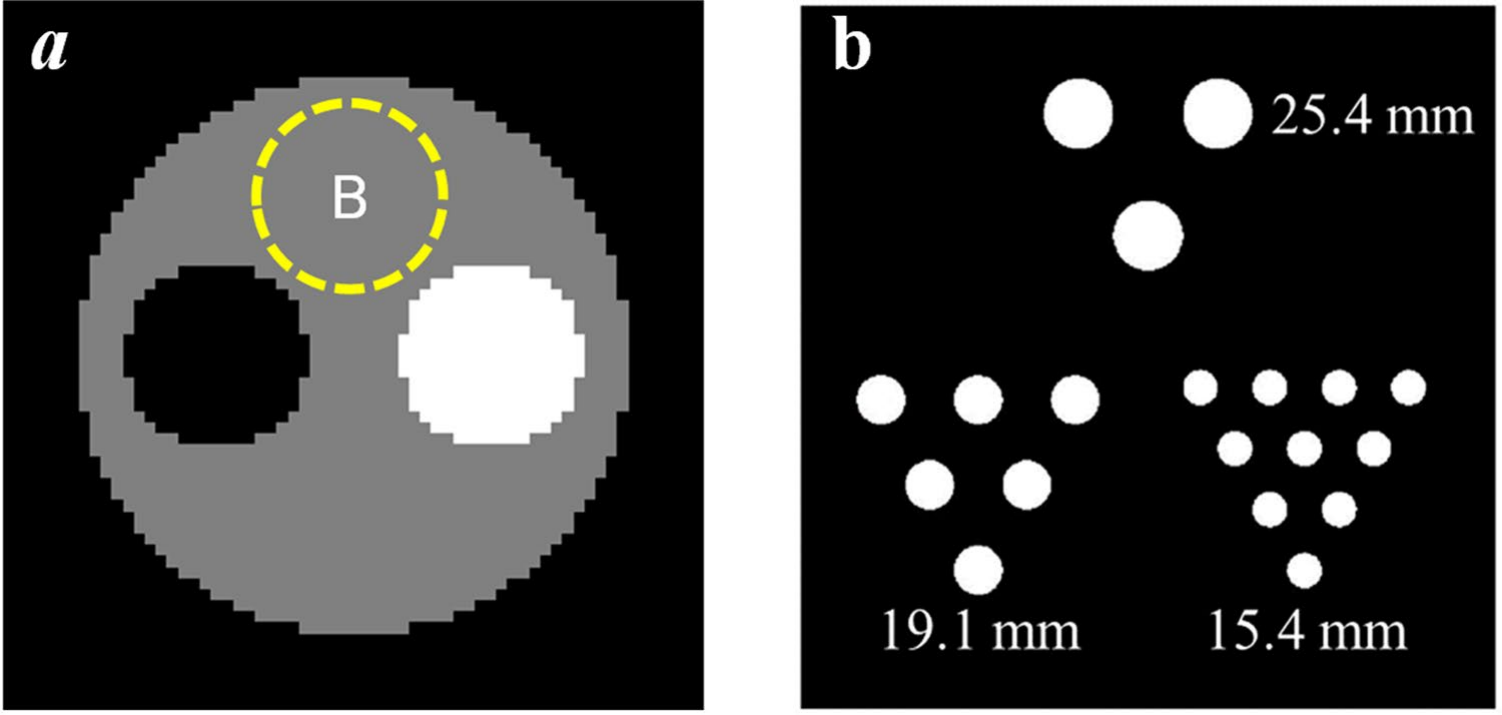
Configurations of (**a**) the contrast phantom and (**b**) the Jaszczak-like rod phantom. The yellow dashed line indicates the area of the background region of interest.

Two digital phantoms with a total Se-75 activity of 2 mCi were used to evaluate the imaging performance; the configurations of the two phantoms are shown in Fig. 5. The contrast phantom was a 30-cm-diameter uniform cylinder containing 10-cm-diameter hot/cold rods placed in the center of the system. The ratio of the activity concentrations for the hot rod:background:cold rod was 2:1:0. A second phantom, a Jaszczak-like rod phantom, was used to assess the reconstructed spatial resolution under varying CTR conditions. This phantom was 25.6 cm in diameter and contained 19 independent rod inserts; each rod was 15-cm long with varying diameters (15.4 mm, 19.1 mm, and 25.4 mm). For the phantom studies, the total acquisition time was 300 s in each trial. The numbers of detected events used in the reconstruction were approximately 5 × 10^6^ for both the contrast and Jaszczak-like rod phantoms. We assumed that only the primary events were included to simplify the conditions and to focus on the influence of the CTR. In the phantom studies, all images were reconstructed using the SOE algorithm. The images were reconstructed into a 64 × 64 matrix with a 6-mm pixel size. All output reconstructed images were post-smoothed by a Gaussian filter with a standard deviation of 0.75.

To enable a quantitative comparison, the coefficient of variation (CV), contrast recovery coefficient (CRC), contrast-to-noise ratio (CNR), and spillover ratio (SOR) were used as image quality metrics to evaluate the system performance under varying CTR conditions. For the contrast phantom, the circular region of interest (ROI) was defined by the size of the hot/cold rods and a background ROI with a 10-cm diameter was placed in the warm background region as shown in Fig. 5.

The coefficient of variation in a warm background (CV_B_) was used to estimate the homogeneity of the radionuclide distribution within the background ROI:

10 $${\mathrm{CV}}_{\mathrm{B}}=\frac{\sigma }{\mu },$$

where *σ* and *μ* denote the standard deviation and the mean value in the background ROI, respectively.

CRC provides information of how accurately the system reproduces the true activity concentration and was defined as

11 $$\mathrm{CRC}=\frac{{C}_{\mathrm{hot}}/{C}_{\mathrm{bkgd}}-1}{{a}_{\mathrm{hot}}/{a}_{\mathrm{bkgd}}-1},$$

where *C*_*hot*_ and *C*_*bkgd*_ are the average counts measured in the reconstructed images in the hot region and background ROIs, respectively. *a*_hot_ and *a*_bkgd_ are the true activity concentration in the hot and background regions, respectively.

CNR measures the signal level in the presence of noise. CNR is defined as

12 $$\mathrm{CNR}=\frac{{C}_{\mathrm{ROI}}-{C}_{\mathrm{bkgd}}}{{\sigma }_{\mathrm{bkgd}}},$$

where *C*_ROI_ (*C*_bkgd_) and *σ*_bkgd_ are the average counts in the ROI (background) and the standard deviation in the background, respectively^33^.

For the cold compartment, the spillover effect caused by the poor spatial resolution can be evaluated via SOR^34^, which is defined as the mean value in the cold ROI *c*_cold_ divided by the mean value in the warm uniform background ROI *c*_bkgd_:

13 $$\mathrm{SOR}=\frac{{C}_{\mathrm{cold}}}{{C}_{\mathrm{bkgd}}}.$$

All reconstructions and analysis calculations were performed in MATLAB software on a 64-bit Linux computer (Intel Core i7-9700 @ 3.00 GHz with 16.0 GB of RAM).

## Results

### System sensitivity and resolution

**Figure 6:**
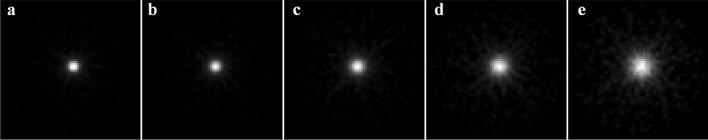
Reconstrcuted image of the point source for the system with full width at half maximum (FWHM) CTRs of (**a**) 0 ps, (**b**) 50 ps, (**c**) 100 ps, (**d**) 150 ps, and (**e**) 200 ps. The image size is 64 × 64 with a pixel size of 4.5 mm × 4.5 mm.

**Figure 7:**
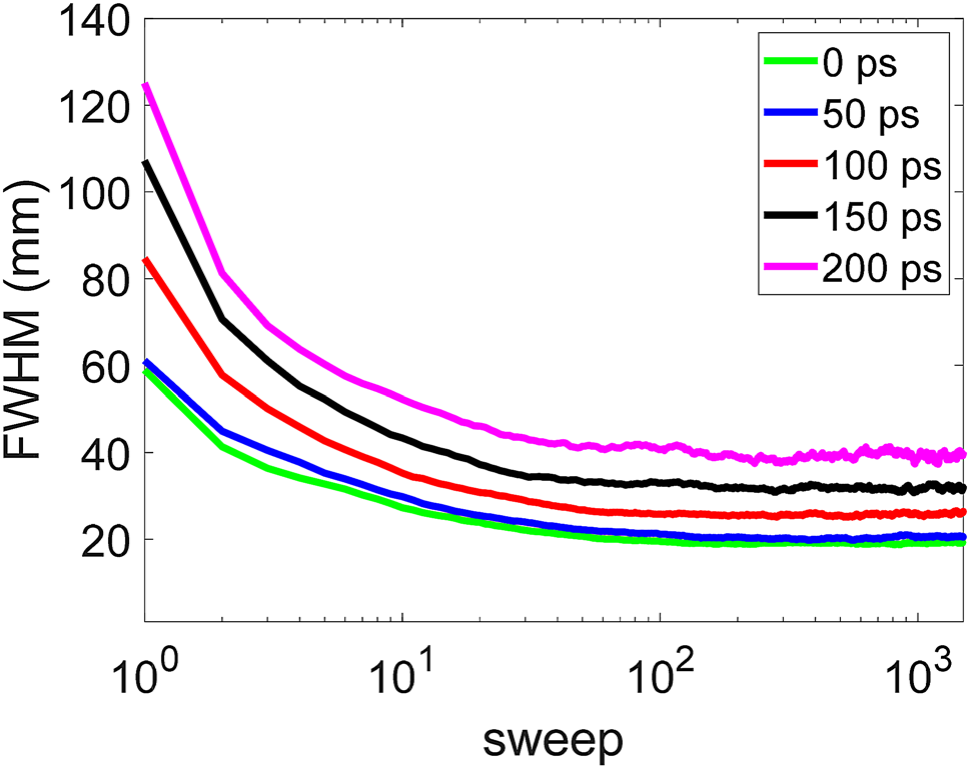
FWHM versus sweep number for the TOF-DuPECT system with different CTRs.

The measured sensitivity of the system was approximately 0.4%. After implementing the dual-energy window, the sensitivity was ~ 0.2%. Figure 6 shows reconstructed images of the point source under different CTR detector conditions. As seen in the figure, the point source located in the central FOV gradually becomes blurrier with increasing CTR. Visually, the point source becomes less concentrated and spreads over the nearby area when the CTR is larger than 150 ps. This figure clearly illustrates the relationship between the spatial resolution and the CTR of the detector. The reconstructed FWHMs as a function of the sweep number for different CTRs are shown in Fig. 7. Following the iterative process, the FWHM values gradually decrease with increasing numbers of sweeps and finally reach a steady state after approximately 200 sweeps. The results show that the FWHM values at 1500 sweeps can reach ~ 20 mm when the CTR is less than 50 ps. For CTRs of 100 ps, 150 ps, and 200 ps, the measured FWHMs are 26.5 mm, 32.2 mm, and 40.1 mm, respectively.

### Effect of the CTR

**Figure 8:**
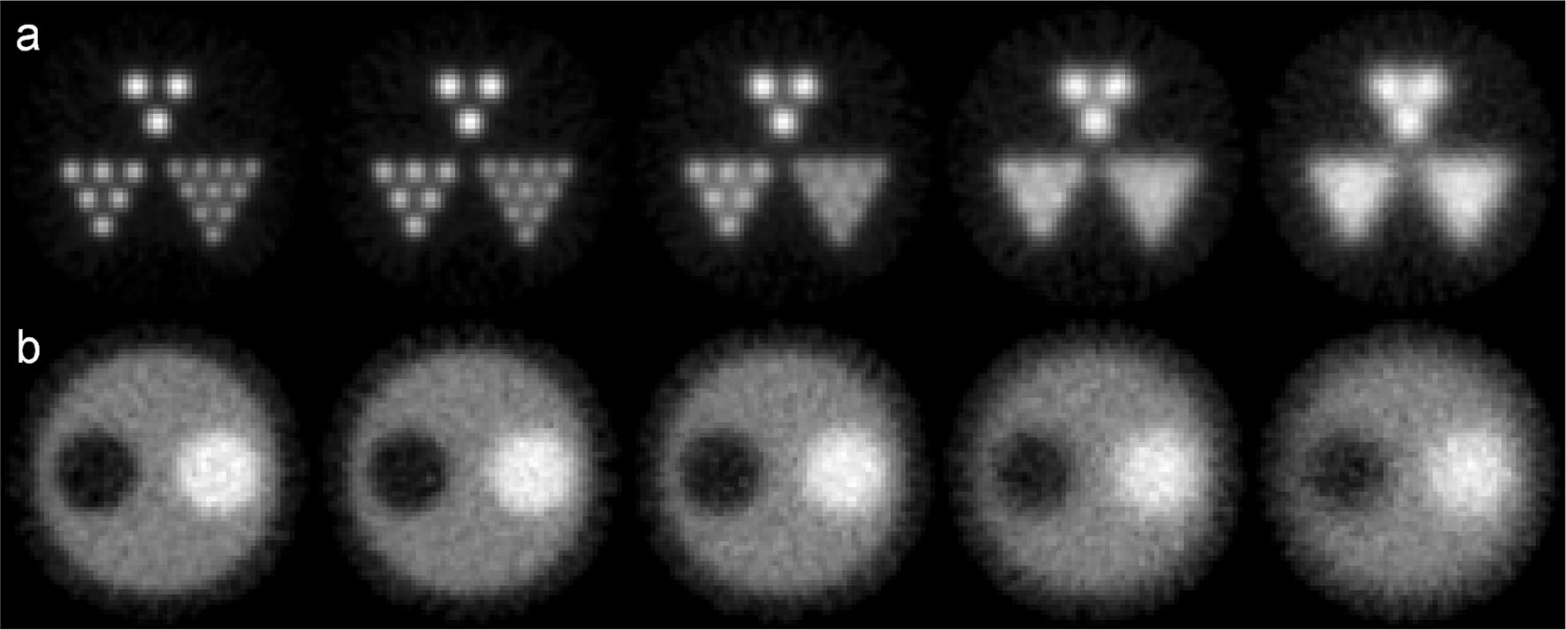
Reconstructed images for (**a**) the Jaszczak-like rod phantom and (**b**) the contrst phantom with CTRs of 0 ps, 50 ps, 100 ps, 150 ps, and 200 ps (from left to right).

**Figure 9:**
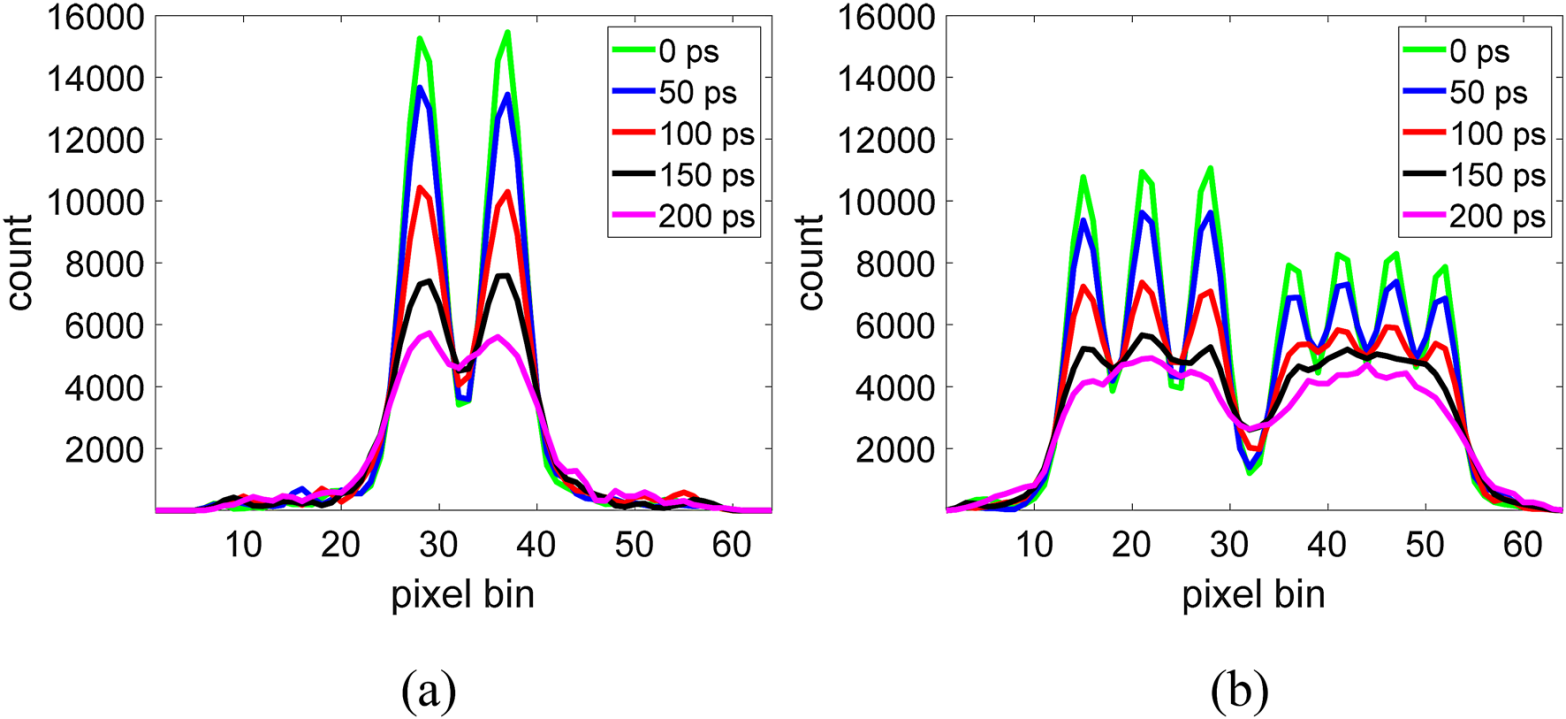
Horizontal profiles of different reconstruction results along (**a**) the 18th row and (**b**) the 35th row of Fig. 8a.

The impacts of the CTR were studied via evaluations of the reconstructed image quality. Figures 8 and 9 show the images reconstructed from TOF-DuPECT with different CTRs and the corresponding horizontal profiles for the Jaszczak-like rod phantom. The resolution obviously improves with decreasing CTR values for the detector. In Fig. 8a, the image obtained with the ideal CTR of 0 ps shows the best performance and the smallest rods with a size of 15.4 mm can be clearly identified. At a CTR of 50 ps, all the hot rods are identifiable; even though the smallest rods become blurry, they are still visible. The 15.4-mm rods remain barely visible until the CTR is greater than 100 ps. In Fig. 9b, the horizontal profile of the 150-ps CTR shows the same result; the peaks of the 15.4-mm rods are no longer identifiable. Observing the changes in the profiles, we find that only the peaks of the largest rods are visible when the CTR is degraded to 200 ps. Figure 8b shows the reconstructed images for the contrast phantom under different CTR conditions. Again, the image with the smallest CTR has the best image quality. With larger CTRs, the images become blurrier and therefore the quality is reduced. This is because the uncertainty in the localization of the source positions increases with the CTR value.

### Image reconstruction performance

**Figure 10:**
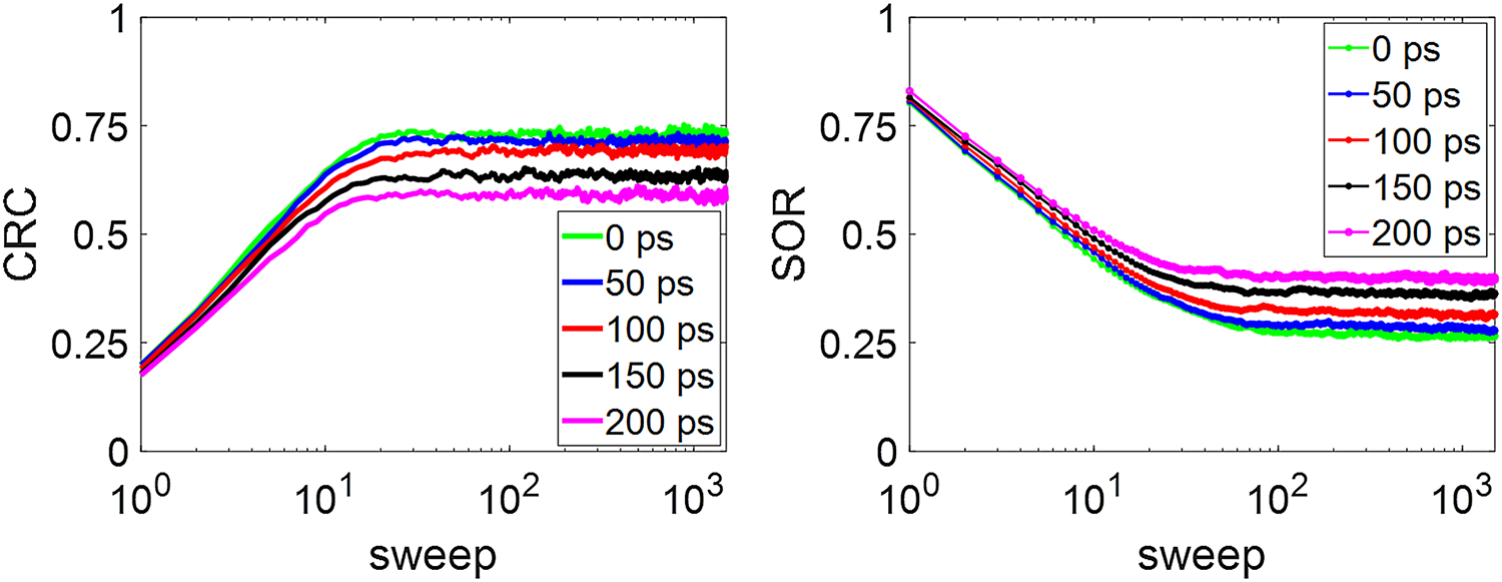
Contrast recovery coefficient (CRC) and spillover ratio (SOR) versus sweep number for the TOF-DuPECT system with different CTRs.

Figure 10 shows the results of CRC and SOR versus the sweep numbers. During the iterative process, the CRC value first increases and then reaches plateaus for each of the CTR values. From the figure, we can see that improvements in the time resolution are favorable to enhancing the CRC performance. SOR shows the opposite trend, where the SOR value decreases until it reaches an equilibrium. SOR increases following the degradation of the CTR, which means that more counts spread into the cold region due to the poor resolution. Table 1 summarizes the measured image quality metrics calculated from the final reconstructed images of the contrast phantom for the different CTRs. Both the CV_B_ and SOR values increase while the CTR degrades. The variability of the reconstructed count density within background ROI increase as the CTR increases. An increase in SOR represents that more counts spread into the cold region and therefore reduce the image quality. It can also be seen that the CRC and CNR values decrease in proportion to the assumed CTR values of the detector. The best CRC and CNR values were 0.733 and 9.784, respectively, when detectors with ideal CTR were applied.

**Table 1:** Measured image quality metrics in reconstructed images of the contrast phantom at varying coincidence time resolutions (CTRs). All indices, the coefficient of variation of the background (CV_B_), contrast recovery coefficient (CRC), contrast-to-noise ratio (CNR) and spillover ratio (SOR), were calculated from the final output image averaging the last 300 of the 1500 sweeps.

CTR	CV_B_	CRC	CNR	SOR
0 ps	0.075	0.733	9.784	0.265
50 ps	0.080	0.712	9.005	0.279
100 ps	0.080	0.690	8.721	0.313
150 ps	0.094	0.637	6.805	0.361
200 ps	0.094	0.588	6.290	0.395

## Discussion

In the point source experiments, we found that the system spatial resolution in the central FOV can achieve an FWHM of approximately 20 mm when the CTR is less than 50 ps. The lack of depth information (e.g., parallax error) and the radionuclide characteristics (e.g., half-lives of the intermediate states) mean that the ideal case with 0-ps CTR cannot achieve a perfectly fine-scale spatial resolution.

Accompanying the degradation of the CTR, the uncertainty in the likelihood emitter position for each hyperbolic response curve derived from TDOA increases, as shown in Fig. 2. This indicates that there exists a strong relationship between the image spatial resolution and the CTR. In this study, we assumed that various CTRs were achievable for a system with the same geometry and composition. In reality, the timing resolution depends on several factors associated with different components of the detector, including the dimensions of the crystal, the performance of light transport, and signal transmissions in the circuit. Current state-of-the-art commercial TOF-PET systems have a working CTR of ~ 210–390 ps^35–38^. As the development of scintillators and photodetectors, CTRs with FWHMs of sub-100 ps have been achieved^39–41^. In 2010, a high time resolution 100-ps CTR was achieved with a detector module coupled with a silicon photomultiplier^42^. More recently, a CTR of 85 ps was realized with 2 mm × 2 mm × 3 mm LSO:Ce crystals and a CTR of 140 ps was achieved for longer 2 mm × 2 mm × 20 mm crystals^40^. However, most of these measurements are based on two single detectors. Additionally, an excellent coincidence resolution below 100 ps FWHM is possible when the photon detection based on Cherenkov light, produced from Cherenkov radiator material, such as lead fluoride (PbF_2_)^43^. The TOF resolution of 143 ps from a whole-body PbF_2_ Cherenkov TOF-PET scanner with multi-layer detector was obtained in a simulation study^44^. With the advancement of technology, the Cherenkov-based detector can drive TOF improvements for imaging devices. Recent research indicates that a CTR of 10 ps may be achievable without a physical barrier, which is promising for the future development of detectors^41^. Consequently, with improved CTRs, the spatial resolution can also be further improved for the proposed TOF-DuPECT system.

In this study, the system sensitivity was also evaluated in the point source tests; the sensitivity of the proposed system is approximately 0.2%, which is approximately one order of magnitude higher than that of clinical SPECT systems (0.01–0.03%)^45^. Compared to clinical PET, the sensitivity of the proposed system is lower by one order of magnitude^45^ owing to the use of a dual-energy window and a smaller time window that restricts a portion of the incident photons.

From the results of the phantom study, in the final images made with the SOE algorithm after running a sufficient number of sweeps, the spatial distribution of the dual-photon emitters could be successfully reconstructed. Consequently, the SOE algorithm appears to be practical and reliable for image reconstructions of the TOF-DuPECT system and can obtain good image quality. We also observed the effect of the CTR in the results of the phantom study. According to the reconstructed results of the Jaszczak-like phantom, a rod size of 19.1 mm can be clearly resolved and a rod size of 15.4 mm can be resolved with careful inspection when the timing resolution reaches 100 ps.

We demonstrated that the concept of TDOA is not only useful in multilateration but also in coincidence image reconstruction. In recent years, a similar system coupled with time information was proposed for image reconstructions of positronium imaging using a trilateration-based algorithm^46,47^. This proposed algorithm is associated with the TOA method, which is a well-known multilateration technique for navigation and positioning. For the three registered photons from ortho-positronium annihilation, the intersection of three circles, which can be determined by the hit positions and hit-times of the photons, corresponds to the annihilation origin point. Similar to our proposed system, the precision of this method relies predominantly on the time resolution of the detector. However, if the CTR can be improved down to a FWHM of 10 ps in the future^41^, the proposed system would be promising and reliable for dual-photon emitter reconstruction with TOF information.

Not all radionuclides that emit two or more cascade photons can be applied to our proposed system. In our preliminary study, Se-75 was used because of its short intermediate state half-life (11.2 ps). However, its long half-life of 120 days may make Se-75 unfavorable for clinical practice, even though it has been used in clinical examinations^26,28^. Other possible radionuclides, such as K-43, have been mentioned in some studies^4,8,10^. However, note that the intermediate state half-life needs to be carefully considered when selecting a suitable dual-photon emitter. For radionuclides with longer intermediate state half-lives, a wider time window is required to detect the two cascade photons and the number of random events will likely increase. In addition, the intermediate state half-life increases the uncertainty in the positioning accuracy and therefore decreases the spatial resolution. For example, a commonly used clinical radionuclide, Indium-111, emits photons at both 171 keV and 245 keV proceeding through an intermediate state with a relatively long half-life of 85 ns; this leads to a high uncertainty in the positioning. Consequently, a proper radionuclide with a short intermediate state half-life for the proposed system is required.

To focus on our exploration of the influence of the CTR, only primary events were included during the reconstruction process in our study. The effects of scatter and attenuation were not considered. Therefore, further work is needed to develop dedicated correction methods for TOF-DuPECT. According to the TDOA theory, the likelihood emitter positions are located along the hyperbolic trajectory derived from the time information. However, the gamma-ray photon paths do not overlap with this hyperbolic curve; this increases the difficulty of effectively estimating the probability of attenuation and scatter at each possible position in space. Recently, a scatter and random correction algorithm based on Bernoulli trials for the SOE algorithm was proposed^48^. Based on this method, if the primary, scatter, and random rates can be pre-calculated prior to the reconstruction, each detected event can be graded and the scatter and random events can be removed during the iterative training. In practice, one can employ energy window-based methods^49,50^ or Monte Carlo-based approaches^51^ to pre-calculate the scatter component when applying our imaging system. Moreover, to further improve the spatial resolution for TOF-DuPECT system, a resolution recovery method can also be considered and implemented via an estimation of the depth of interaction in the detector or by modeling the uncertainty of the timing. In 2016, Andreyev proposed a modified version of the SOE algorithm to model the probability distributions of the measured energy and the interaction locations in the detectors of a Compton camera^52^. This modified SOE algorithm enables images to be reconstructed with a resolution recovery option with little additional computational cost. For a low-count condition, reconstructed images using the SOE algorithm are often noisy and inhomogeneous in density. Our previous study developed a noise smoothing origin ensemble algorithm based on local filtering that can be used to reduce the image noise and further improve the image quality under low-count statistics^53^. Further improvements to our proposed TOF-DuPECT system may be achieved when these approaches are incorporated into the SOE reconstruction.

In the study, the average reconstruction time per sweep is approximately 20.1 s and 9.6 min for the point source and phantom studies, respectively. The computation cost correlates with the size of list-mode data, and therefore, reconstruction in phantom studies needs more computational time. During the iteration process, the total memory usages were approximately 158.7 MB and 1.95 GB for the point and phantom experiments, respectively. Most of the memory is consumed in the process of loading and temporarily storing the list-mode data. Our current reconstruction algorithm was implemented with MATLAB and has not yet been optimized. Further efforts should be made to accelerate the computational time using GPU and an optimal projector, allowing to reconstruct images in real time.

To summarize, we demonstrated the viability of TOF-DuPECT and performed a preliminary image quality analysis on images reconstructed using the SOE algorithm. The performances for varying CTR values were also evaluated. Unlike convention imaging systems, the proposed system incorporates TDOA information and can be applied to a variety of dual-photon emitters with short intermediate state half-lives. Based on our findings, the performance is primarily restricted by the timing resolution of the imaging system. However, with the advancement of technology, the proposed system is promising and worthy of continued research. Future studies are needed to improve the image quality and to facilitate further development of quantitative imaging techniques such as attenuation and scatter corrections for the proposed system.

## Conclusions

The TOF-DuPECT system incorporating the TDOA technique is feasible and has potential. The distribution of dual-photon emitters can be reconstructed in terms of the arrival times and hit positions using the SOE algorithm. The performance of the proposed system was estimated using Monte Carlo simulations. We observed that 15.4-mm rods could be resolved when the timing resolution reached 100 ps. The most important advantage of TOF-DuPECT imaging over SPECT is that it exhibits high sensitivity with acceptable spatial resolution. This technique could be extended to nuclides that do not emit positrons even though the spatial resolution of the system is primarily limited by the CTR of the detectors. Further phantom studies and correction technique development are required for the TOF-DuPECT system including scatter correction and attenuation correction studies.
